# Inhibitory Effects of Cytosolic Ca^2+^ Concentration by Ginsenoside Ro Are Dependent on Phosphorylation of IP_3_RI and Dephosphorylation of ERK in Human Platelets

**DOI:** 10.1155/2015/764906

**Published:** 2015-08-19

**Authors:** Hyuk-Woo Kwon, Jung-Hae Shin, Dong-Ha Lee, Hwa-Jin Park

**Affiliations:** ^1^Department of Biomedical Laboratory Science, College of Biomedical Science and Engineering, Inje University, 197 Inje-ro, Gimhae, Gyungnam 621-749, Republic of Korea; ^2^Department of Biomedical Laboratory Science, Korea Nazarene University, 48 Wolbong-ro, Seobuk gu, Cheonan, Chungnam 331-778, Republic of Korea

## Abstract

Intracellular Ca^2+^ ([Ca^2+^]_*i*_) is platelet aggregation-inducing molecule and is involved in activation of aggregation associated molecules. This study was carried out to understand the Ca^2+^-antagonistic effect of ginsenoside Ro (G-Ro), an oleanane-type saponin in *Panax ginseng*. G-Ro, without affecting leakage of lactate dehydrogenase, dose-dependently inhibited thrombin-induced platelet aggregation, and the half maximal inhibitory concentration was approximately 155 *μ*M. G-Ro inhibited strongly thrombin-elevated [Ca^2+^]_*i*_, which was strongly increased by A-kinase inhibitor Rp-8-Br-cAMPS compared to G-kinase inhibitor Rp-8-Br-cGMPS. G-Ro increased the level of cAMP and subsequently elevated the phosphorylation of inositol 1, 4, 5-triphosphate receptor I (IP_3_RI) (Ser^1756^) to inhibit [Ca^2+^]_*i*_ mobilization in thrombin-induced platelet aggregation. Phosphorylation of IP_3_RI (Ser^1756^) by G-Ro was decreased by PKA inhibitor Rp-8-Br-cAMPS. In addition, G-Ro inhibited thrombin-induced phosphorylation of ERK 2 (42 kDa), indicating inhibition of Ca^2+^ influx across plasma membrane. We demonstrate that G-Ro upregulates cAMP-dependent IP_3_RI (Ser^1756^) phosphorylation and downregulates phosphorylation of ERK 2 (42 kDa) to decrease thrombin-elevated [Ca^2+^]_*i*_, which contributes to inhibition of ATP and serotonin release, and p-selectin expression. These results indicate that G-Ro in *Panax ginseng* is a beneficial novel Ca^2+^-antagonistic compound and may prevent platelet aggregation-mediated thrombotic disease.

## 1. Introduction

The full platelet aggregation by physiological agonists (i.e., thrombin, ADP, and collagen) is absolutely essential for the formation of a hemostatic plug when normal blood vessels are injured. This physiological event is underlied by an elevation of cytosolic free Ca^2+^ level ([Ca^2+^]_*i*_) and can also cause circulatory disorders, such as thrombosis, atherosclerosis, and myocardial infarction [1–3]. Accordingly, inhibiting [Ca^2+^]_*i*_-elevation by platelet agonists is a promising approach for the prevention of thrombosis. Elevation of [Ca^2+^]_*i*_ by agonists is dependent on the mobilization from intracellular Ca^2+^ stores (endoplasmic reticulum or dense tubular system) and the influx of extracellular Ca^2+^ across plasma membrane (PM) [1–3]. It is known that thrombin, a platelet agonist, stimulates platelet aggregation by binding to the Gq-coupled proteinase-activated receptor, which is involved in activating phospholipase C-*β* (PLC-*β*). Activated PLC-*β* hydrolyzes phosphatidylinositol 4, 5-bisphosphate (PIP_2_) to inositol 1, 4, 5-trisphosphate (IP_3_) and diacylglycerol (DG) [4–7]. Moreover, IP_3_ mobilizes cytosol free Ca^2+^ ([Ca^2+^]_*i*_) from dense tubular system (DTS) by binding to IP_3_ receptor type I (IP_3_RI). The increased [Ca^2+^]_*i*_ activates both the Ca^2+^/calmodulin-dependent phosphorylation of myosin light chain (20 kDa) and the DG-dependent phosphorylation of pleckstrin (47 kDa) to induce granule secretion (i.e., dense body and *α*-granule) and platelet aggregation [8, 9]. The Ca^2+^-antagonistic effects of cAMP and cGMP are mediated* via* cAMP- and cGMP-dependent protein kinase (PKA and PKG), which phosphorylates substrate protein, IP_3_RI. The action of IP_3_RI is inhibited by its phosphorylation, and IP_3_RI phosphorylation is involved in inhibition of [Ca^2+^]_*i*_ mobilization [10–12]. Therefore, phosphorylating IP_3_RI is very useful for evaluating the Ca^2+^-antagonistic effect of substances or compounds. With regard to the influx of extracellular Ca^2+^ across PM, it is well known that phosphatidylinositol 3-kinase and phosphatidylinositol 4-kinase (PI3K and PI4K) and extracellular signal-regulated kinase (ERK) are involved in the entry of extracellular Ca^2+^ across PM by activating the Ca^2+^-permeable channel protein and human transient receptor potential channel (hTrp1) coupling with IP_3_R type II (IP_3_RII) [13–18].

**Figure 1:**
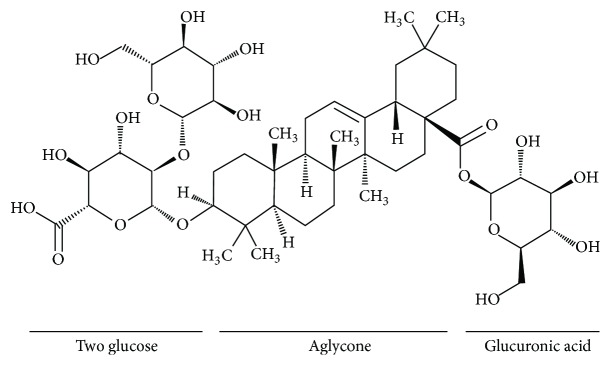
Chemical structure of ginsenoside Ro. Ginsenoside Ro (G-Ro), an oleanane-type saponin, is contained in* Panax ginseng* Meyer [19, 20] and is composed of oleanolic acid as aglycone and two glucose and one glucuronic acid as sugar component [19].

Ginseng, the root of* Panax ginseng *Meyer, has been used frequently in traditional oriental medicine and is known to have various pharmacological activities such as anti-inflammatory action, antioxidation, antitumor, antidiabetes, and antihepatotoxicity [21, 22]. In recent, it is reported that Korean red ginseng has an effect on cardiovascular disease, which is characterized with regard to reduction of blood pressure and arterial stiffness by inhibition of Rho kinase [23], anticoagulation by prolonged prothrombin and activated partial thromboplastin time [24], endothelium relaxation by nitric oxide-cGMP pathway [25], and inhibition of hypercholesterolemia-induced platelet aggregation [26]. In our previous report, we demonstrated that total saponin from Korean red ginseng (TSKRG) is a beneficial traditional oriental medicine in platelet-mediated thrombotic disease* via* suppression of cyclooxygenase-1 (COX-1) and thromboxane A_2_ synthase (TXAS) to inhibit production of thromboxane A_2_ (TXA_2_) [27]. It is reported that ginsenoside Ro (G-Ro, Figure 1) has no inhibitory effect on collagen-elevated [Ca^2+^]_*i*_ [28]. However, in this study, we showed that G-Ro attenuates thrombin-elevated [Ca^2+^]_*i*_ *via* cAMP-dependent IP_3_RI phosphorylation and dephosphorylation of ERK in human platelets. This study provides novel information for antiplatelet effects of G-Ro in ginseng.

## 2. Materials and Methods

### 2.1. Materials

Ginsenoside Ro (G-Ro, Figure 1) was obtained from Ambo Institute (Daejeon, Korea). Thrombin was purchased from Chrono-Log Corporation (Havertown, PA, USA). ATP assay kit was purchased from Biomedical Research Service Center (Buffalo, NY, USA). Serotonin ELISA kit was purchased from Labor Diagnostika Nord GmbH & Corporation (Nordhorn, Germany). A-kinase inhibitor Rp-8-Br-cAMPS, G-kinase inhibitor Rp-8-Br-cGMPS, and 2-acetoxymethyl (Fura 2-AM) were obtained from Sigma Chemical Corporation (St. Louis, MO, USA). Lactate dehydrogenase (LDH) cytotoxicity assay kit, cAMP enzyme immunoassay (EIA) kit, and cGMP enzyme immunoassay (EIA) kit were obtained from Cayman Chemical (Ann Arbor, MI, USA). Anti-IP_3_-receptor type I, antiphosphor-IP_3_-receptor type I (Ser^1756^), anti-ERK (1/2), antiphosphor-ERK (1/2), anti-rabbit IgG-horseradish peroxidase conjugate (HRP), and lysis buffer were obtained from Cell Signaling (Beverly, MA, USA). Anti-*β*-actin was obtained from Santa Cruz Biotechnology (Santa Cruz, CA, USA). Mouse monoclonal to CD62P (p-selectin) antibody was purchased from Biolegend (San Diego, CA, USA). Polyvinylidene difluoride (PVDF) membrane was from GE Healthcare (Piscataway, New Jersey, USA). Enhanced chemiluminescence solution (ECL) was from GE Healthcare (Chalfont St. Giles, Buckinghamshire, UK).

### 2.2. Preparation of Washed Human Platelets

Human platelet-rich plasma (PRP) anticoagulated with acid-citrate-dextrose solution (0.8% citric acid, 2.2% sodium citrate, and 2.45% glucose) was obtained from Korean Red Cross Blood Center (Changwon, Korea). PRP was centrifuged for 10 min at 125 g to remove a few red blood cells and white blood cells and was centrifuged for 10 min at 1,300 g to obtain the platelet pellets. The platelets were washed twice with washing buffer (138 mM NaCl, 2.7 mM KCl, 12 mM NaHCO_3_, 0.36 mM NaH_2_PO_4_, 5.5 mM glucose, and 1 mM Na_2_EDTA, pH 6.5). The washed platelets were then resuspended in suspension buffer (138 mM NaCl, 2.7 mM KCl, 12 mM NaHCO_3_, 0.36 mM NaH_2_PO_4_, 0.49 mM MgCl_2_, 5.5 mM glucose, and 0.25% gelatin, pH 6.9) to a final concentration of 5 × 10^8^/mL. All of the above procedures were carried out at 25°C to avoid platelet aggregation from any effect of low temperature. The Korea National Institute for Bioethics Policy Public Institutional Review Board (Seoul, Korea) approved these experiments (PIRB12-072).

### 2.3. Measurement of Platelet Aggregation

Human washed platelets (10^8^/mL) were preincubated for 3 min at 37°C in the presence of 2 mM exogenous CaCl_2_ with or without substances and then stimulated with thrombin (0.05 U/mL) for 5 min. Aggregation was monitored using an aggregometer (Chrono-Log, Corporation) at a constant stirring speed of 1,000 rpm. Each aggregation rate was calculated as an increase in light transmission. The suspension buffer was used as the reference (transmission 0). G-Ro was dissolved in platelet suspension buffer (pH 6.9).

### 2.4. Lactate Dehydrogenase Activity Assay

Human platelet cytotoxicity was determined by the leakage of lactate dehydrogenase (LDH) from cytosol. Human washed platelets (10^8^/mL) were incubated for 5 min at 37°C with various concentrations of G-Ro and then centrifuged at room temperature for 2 min at 12,000 g. The supernatant was measured by LDH assay kit (Cayman Chemical) at an optical density of 490 nm. LDH leakage is expressed as the percentage of the total enzyme activity in platelets completely lysed with 0.1% Triton X-100.

### 2.5. Determination of Cytosolic Free Ca^2+^ ([Ca^2+^]_*i*_)

PRP was incubated with 5 *μ*M Fura 2-AM at 37°C for 60 min. Because Fura 2-AM is light sensitive, the tube containing the PRP was covered with aluminum foil during loading. The Fura 2-loaded washed platelets were prepared using the procedure described above and 10^8^ platelets/mL were preincubated for 3 min at 37°C with or without G-Ro in the presence of 2 mM CaCl_2_ and then stimulated with thrombin (0.05 U/mL) for 5 min for evaluation of [Ca^2+^]_*i*_. Fura 2 fluorescence was measured with a spectrofluorometer (SFM 25, BioTeck Instrument, Italy) with an excitation wavelength that was changed every 0.5 sec from 340 to 380 nm; the emission wavelength was set at 510 nm. The [Ca^2+^]_*i*_ values were calculated using the method of Grynkiewicz [29].

### 2.6. Measurement of cAMP and cGMP

Washed human platelets (10^8^/mL) were preincubated for 3 min at 37°C with or without various concentrations of G-Ro in the presence of 2 mM CaCl_2_ and then stimulated with thrombin (0.05 U/mL) for 5 min for platelet aggregation. The aggregation was terminated by the addition of 80% ice-cold ethanol. cAMP and cGMP were measured with Synergy HT Multi-Model Microplate Reader (BioTek Instruments, Winooski, VT, USA).

### 2.7. Western Blot for Analysis of IP_3_RI and ERK Phosphorylation

Washed platelets (10^8^/mL) were preincubated with or without G-Ro in the presence of 2 mM CaCl_2_ for 3 min and then stimulated with thrombin (0.05 U/mL) for 5 min at 37°C. The reactions were terminated by adding an equal volume (250 *μ*L) of lysis buffer (20 mM Tris-HCl, 150 mM NaCl, 1 mM Na_2_EDTA, 1 mM EGTA, 1% Triton X-100, 2.5 mM sodium pyrophosphate, 1 mM serine/threonine phosphatase inhibitor *β*-glycerophosphate, 1 mM ATPase, alkaline and acid phosphatase, protein phosphotyrosine phosphatase inhibitor Na_3_VO_4_, 1 *μ*g/mL serine and cysteine protease inhibitor leupeptin, and 1 mM serine protease and acetylcholinesterase inhibitor phenylmethanesulfonyl fluoride, pH 7.5). Platelet lysates containing the same protein (15 *μ*g) were used for analysis. Protein concentrations were measured by using bicinchoninic acid (BCA) protein assay kit (Pierce Biotechnology, USA). The effects of G-Ro on IP_3_RI phosphorylations were analyzed by western blotting. A 6–8% SDS-PAGE (1.5 mm gel) was used for electrophoresis and a PVDF membrane was used for protein transfer from the gel. The dilutions for anti-IP_3_RI, antiphosphor-IP_3_RI (Ser^1756^), anti-ERK (1/2), antiphosphor-ERK (1/2), and anti-rabbit IgG-HRP were 1 : 1000, 1 : 1000, 1 : 1000, 1 : 1000, and 1 : 10000, respectively. The membranes were visualized using ECL. Blots were analyzed by using the Quantity One, Version 4.5 (BioRad, Hercules, CA, USA).

### 2.8. Determination of ATP and Serotonin Release

Washed platelets (10^8^/mL) were preincubated for 3 min at 37°C with or without G-Ro and other reagents in the presence of 2 mM CaCl_2_ and then stimulated with thrombin (0.05 U/mL). The reaction was terminated and centrifuged with 200 g at 4°C for 10 min, and supernatants were used for the assay of ATP and serotonin release. ATP release was measured in a luminometer (BioTek Instruments) using an ATP assay kit. Serotonin release was measured with a Synergy HT Multi-Model Microplate Reader (BioTek Instruments, Winooski, VT, USA) using serotonin ELISA kit.

### 2.9. Determination of p-Selectin Release

Washed human platelets (10^8^/mL) were preincubated for 3 min at 37°C with or without substances in the presence of 2 mM CaCl_2_ and then stimulated with thrombin (0.05 U/mL). The platelets were reconstituted by ice-cold phosphate-buffered saline (*PBS, pH 7.4*) 250 *μ*L and cells were incubated with Alexa Fluor 488 anti-human CD62P (10 *μ*L) in PBS (pH 7.4), containing 0.09% sodium azide and 0.2% bovine serum albumin (BSA) for 60 min at 4°C in the dark room. Next, platelets were washed three times by ice-cold PBS and resuspended by 0.5% paraformaldehyde in PBS. Alexa Fluor 488 anti-human CD62P binding to platelets was determined using flow cytometry (BD Biosciences, San Diego, CA, USA) and data were analyzed using CellQuest software.

### 2.10. Statistical Analyses

The experimental results are expressed as the mean ± standard deviation accompanied by the number of observations. Data were assessed by analysis of variance (ANOVA). If this analysis indicated significant differences among the group means, then each group was compared by the Newman-Keuls method. Statistical analysis was performed according to the SPSS 21.0.0 (SPSS, Chicago, IL, USA). *p* < 0.05 was considered to be statistically significant.

## 3. Results

### 3.1. Effects of G-Ro on Thrombin-Induced Human Platelet Aggregation

**Figure 2:**
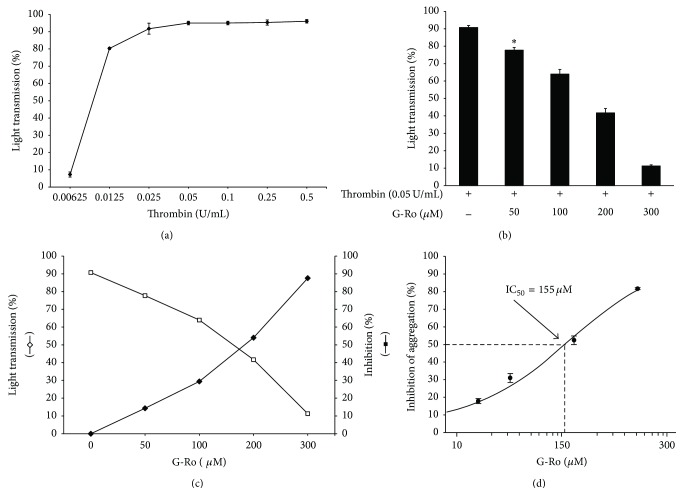
Effects of G-Ro on thrombin-induced human platelet aggregation. (a) The concentration threshold of thrombin on human platelet aggregation. (b) Effects of G-Ro on thrombin-induced human platelet aggregation. (c) The inhibitory effects of G-Ro on thrombin-induced human platelet aggregation. (d) The IC_50_ value of G-Ro in thrombin-stimulated human platelet aggregation. Measurement of platelet aggregation was carried out as described in “Section 2.” The rate of inhibition by G-Ro was recorded as the percentage of the thrombin-induced aggregation rate. The IC_50_ value of G-Ro was calculated according to the 4-parameter log fit method. The data are expressed as the mean ± standard deviation (*n* = 4). ^*∗*^ *p* < 0.05 versus the thrombin-stimulated human platelets.

The concentration of thrombin-induced maximal human platelet aggregation was approximately 0.05 U/mL (Figure 2(a)). Therefore, thrombin (0.05 U/mL) was used as the human platelet agonist in this study. When washed human platelets (10^8^/mL) were activated with thrombin, the aggregation rate was increased up to 90.7 ± 1.2%. However, various concentrations of G-Ro (50, 100, 200, and 300 *μ*M) dose-dependently reduced thrombin-stimulated platelet aggregation (Figures 2(b) and 2(c)), and the half-maximal inhibitory concentration (IC_50_) was approximately 155 *μ*M (Figure 2(d)).

### 3.2. Effects of Cytotoxicity of G-Ro to Human Platelets

**Figure 3:**
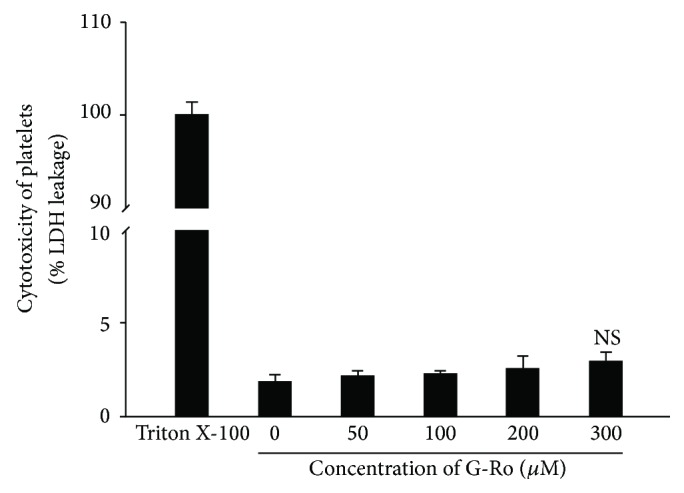
Effects of G-Ro on cytotoxicity. Measurement of cytotoxicity was carried out as described in “Section 2.” For a positive control, 0.1% Triton X-100 was used to treat platelets. The data are expressed as the mean ± standard deviation (*n* = 4). NS, not significant versus without G-Ro, control.

Cytotoxicity of drugs is evaluated by cytosolic LDH leakage, which is different from platelet aggregation or granule secretion by platelet agonists [30–32]. Therefore, we investigate the effect of G-Ro on cytotoxicity to human platelets. When human platelets (10^8^/mL) were treated by membrane detergent Triton X-100 as a positive control, LDH was potently released (Figure 3). However, G-Ro (50 to 300 *μ*M) that inhibited thrombin-induced platelet aggregation did not release LDH as compared to that by Triton X-100. LDH leakage by various concentrations of G-Ro (50, 100, 200, and 300 *μ*M) was 2.1% (at 50 *μ*M of G-Ro), 2.2% (at 100 *μ*M of G-Ro), 2.5% (at 200 *μ*M of G-Ro), and 2.9% (at 300 *μ*M of G-Ro), respectively, which were not significantly different from that (1.8%) by resting platelets (Figure 3).

### 3.3. Effects of G-Ro on Elevation of Cytosolic Free Ca^2+^ ([Ca^2+^]_*i*_)

**Figure 4:**
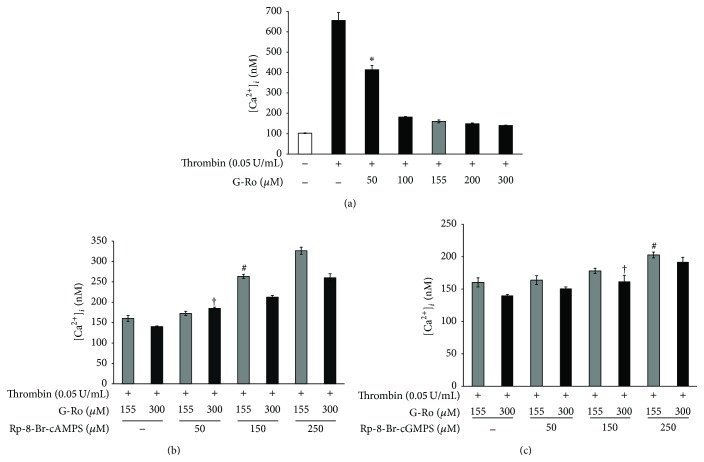
Effects of G-Ro on thrombin-induced [Ca^2+^]_*i*_ mobilization. (a) Effects of G-Ro on [Ca^2+^]_*i*_ level in thrombin-induced platelet aggregation. (b) Effects of G-Ro on [Ca^2+^]_*i*_ level in the presence of A-kinase inhibitor (Rp-8-Br-cAMPS). (c) Effects of G-Ro on [Ca^2+^]_*i*_ level in the presence of G-kinase inhibitor (Rp-8-Br-cGMPS). [Ca^2+^]_*i*_ was determined as described in “Section 2.” The data are expressed as the mean ± standard deviation (*n* = 4). ^*∗*^ *p* < 0.05 versus the thrombin-stimulated human platelets, ^#^ *p* < 0.05 versus the thrombin-stimulated human platelets in the presence of G-Ro (155 *μ*M), and ^†^ *p* < 0.05 versus the thrombin-stimulated human platelets in the presence of G-Ro (300 *μ*M).

Because [Ca^2+^]_*i*_ is essential for platelet activation by agonists, we investigated the effect of G-Ro on Ca^2+^-antagonistic activity. As shown in Figure 4(a), thrombin increased [Ca^2+^]_*i*_ level from 102.4 ± 0.5 nM, the basal level, to 655.7 ± 38.7 nM (Figure 4(a)). However, G-Ro dose-dependently (50 to 300 *μ*M) decreased thrombin-elevated [Ca^2+^]_*i*_ (Figure 4(a)). If G-Ro-mediated decrease of [Ca^2+^]_*i*_ level resulted from cAMP/PKA-pathway or cGMP/PKG-pathway, the G-Ro-reduced [Ca^2+^]_*i*_ level would be increased by inhibitors of PKA or PKG. Accordingly, we investigated the effects of PKA inhibitor Rp-8-Br-cAMPS and PKG inhibitor Rp-8-Br-cGMPS on IC_50_ (155 *μ*M) and 300 *μ*M of G-Ro-reduced [Ca^2+^]_*i*_ level, which is to observe an apparent elevation of [Ca^2+^]_*i*_ by their inhibitors. As shown in Figure 4(b), the PKA inhibitor Rp-8-Br-cAMPS (50 to 250 *μ*M) dose-dependently increased the [Ca^2+^]_*i*_ level as compared with those (160.3 ± 7.1, 140.1 ± 1.8 nM) by G-Ro (155, 300 *μ*M) in the thrombin-induced platelet aggregation (Figure 4(b)). As shown in Table 1, Rp-8-Br-cAMPS (250 *μ*M) increased G-Ro-decreased (155, 300 *μ*M) [Ca^2+^]_*i*_ levels to 103.6% and 85.8%, respectively. But Rp-8-Br-cGMPS (250 *μ*M) increased G-Ro-decreased (155, 300 *μ*M) [Ca^2+^]_*i*_ levels to 26.4% and 36.9%, respectively (Table 1, Figure 4(c)).

### 3.4. Effects of G-Ro on cAMP and cGMP Production

**Figure 5:**
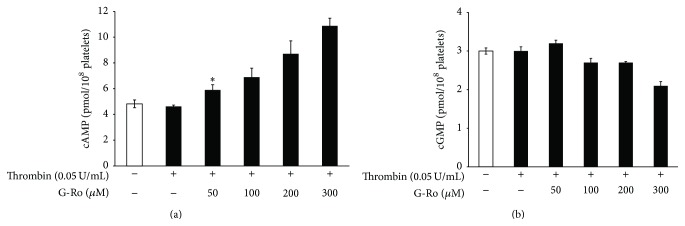
Effects of G-Ro on cAMP and cGMP production. (a) Effects of G-Ro on cAMP production in thrombin-induced platelets. (b) Effects of G-Ro on cGMP production in thrombin-induced platelets. cAMP and cGMP were determined as described in “Section 2.” The data are expressed as the mean ± standard deviation (*n* = 4). ^*∗*^ *p* < 0.05 versus the thrombin-stimulated human platelets.

Because it was confirmed that inhibition of [Ca^2+^]_*i*_ level by G-Ro is dependent on cAMP/PKA and cGMP/PKG, next, we investigated whether G-Ro increases cAMP and cGMP production in thrombin-induced platelet aggregation. As shown in Figure 5(a), thrombin weakly decreased cAMP level, but G-Ro (50 to 300 *μ*M) dose-dependently increased thrombin-attenuated cAMP level. With regard to cGMP, thrombin did not change cGMP level as compared with that of basal level; on the contrary, cGMP appears to be decreased by G-Ro (Figure 5(b)).

### 3.5. Effects of G-Ro on Inositol 1, 4, 5-Trisphosphate Receptor Type I (IP_3_RI) (Ser^1756^) Phosphorylation

**Figure 6:**
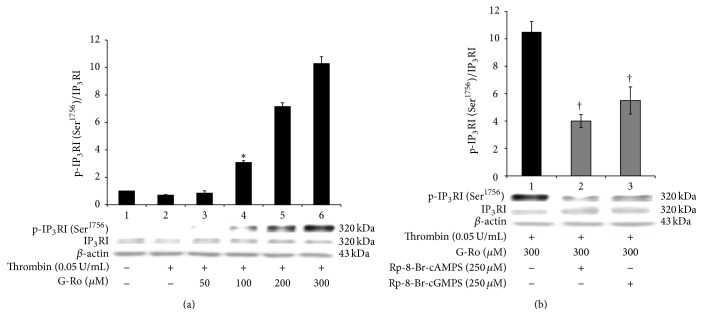
Effects of G-Ro on inositol 1, 4, 5-trisphosphate receptor type I (IP_3_RI) (Ser^1756^) phosphorylation. (a) Effects of G-Ro on IP_3_RI (Ser^1756^) phosphorylation. Lane 1, unstimulated platelets (base); Lane 2, thrombin (0.05 U/mL); Lane 3, thrombin (0.05 U/mL) + G-Ro (50 *μ*M); Lane 4, thrombin (0.05 U/mL) + G-Ro (100 *μ*M); Lane 5, thrombin (0.05 U/mL) + G-Ro (200 *μ*M); Lane 6, thrombin (0.05 U/mL) + G-Ro (300 *μ*M). (b) Effects of G-Ro on IP_3_RI (Ser^1756^) phosphorylation in the presence of A-kinase inhibitor (Rp-8-Br-cAMPS) and G-kinase inhibitor (Rp-8-Br-cGMPS). Lane 1, thrombin (0.05 U/mL) + G-Ro (300 *μ*M); Lane 2, thrombin (0.05 U/mL) + G-Ro (300 *μ*M) + Rp-8-Br-cAMPS (250 *μ*M); Lane 3, thrombin (0.05 U/mL) + G-Ro (300 *μ*M) + Rp-8-Br-cGMPS (250 *μ*M). Western blotting was performed as described in “Section 2.” The data are expressed as the mean ± standard deviation (*n* = 4). ^*∗*^ *p* < 0.05 versus the thrombin-stimulated human platelets; ^†^ *p* < 0.05 versus the thrombin-stimulated human platelets in the presence of G-Ro (300 *μ*M).

G-Ro (50 to 300 *μ*M) dose-dependently increased phosphorylation [IP_3_R (Ser^1756^)] of IP_3_RI (Ser^1756^) and the ratio of p-IP_3_RI (Ser^1756^) to IP_3_RI in thrombin-induced human platelet aggregation (Figure 6(a) Lanes 3 to 6). To know whether IP_3_RI (Ser^1756^) phosphorylation by G-Ro was resulted from cAMP/PKA-pathway or cGMP/PKG-pathway, we investigated an apparent inhibitory effect of their inhibitors on IP_3_RI (Ser^1756^) phosphorylation by 300 *μ*M of G-Ro that potently stimulates the phosphorylation of IP_3_RI (Ser^1756^). As shown in Figure 6(b), Lane 2, PKA inhibitor Rp-8-Br-cAMPS decreased G-Ro-increased p-IP_3_RI (Ser^1756^) in thrombin-induced platelet aggregation. PKG inhibitor Rp-8-Br-cGMPS also decreased G-Ro-induced p-IP_3_RI (Ser^1756^) (Figure 6(b), Lane 3). But inhibitory degree by PKG inhibitor Rp-8-Br-cGMPS was lower as compared with that by PKA inhibitor Rp-8-Br-cAMPS.

### 3.6. Effects of G-Ro on Dephosphorylation of ERK

**Figure 7:**
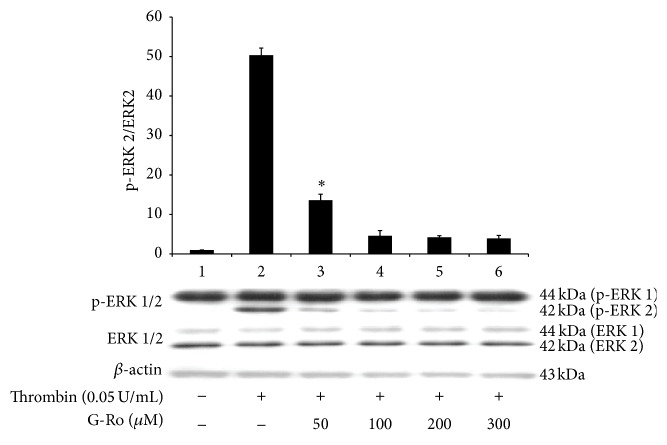
Effects of G-Ro on dephosphorylation of ERK. Lane 1, unstimulated platelets (base); Lane 2, thrombin (0.05 U/mL); Lane 3, thrombin (0.05 U/mL) + G-Ro (50 *μ*M); Lane 4, thrombin (0.05 U/mL) + G-Ro (100 *μ*M); Lane 5, thrombin (0.05 U/mL) + G-Ro (200 *μ*M); Lane 6, thrombin (0.05 U/mL) + G-Ro (300 *μ*M). Western blotting was performed as described in “Section 2.” The data are expressed as the mean ± standard deviation (*n* = 4). ^*∗*^ *p* < 0.05 versus the thrombin-stimulated human platelets.

It is known that Ca^2+^ mobilized from DTS (ER) is involved in phosphorylation of PI3K and ERK to influx extracellular Ca^2+^ [13, 14, 17, 18]. Here, we investigated the effect of G-Ro on dephosphorylation of ERK (1/2). As shown in Figure 7, in unstimulated platelets, ERK 1 (44 kDa) phosphorylation only was observed (Figure 7, Lane 1). Thrombin potently phosphorylated both ERK 1 (44 kDa) and ERK 2 (42 kDa) (Figure 7, Lane 2). However, G-Ro (50 to 300 *μ*M) dose-dependently inhibited thrombin-induced phosphorylation of ERK 2 (42 kDa), and the ratio of p-ERK 2 to ERK 2 (42 kDa) (Figure 7, Lanes 3 to 6).

### 3.7. Effects of G-Ro on ATP and Serotonin Release from Dense Body

**Figure 8:**
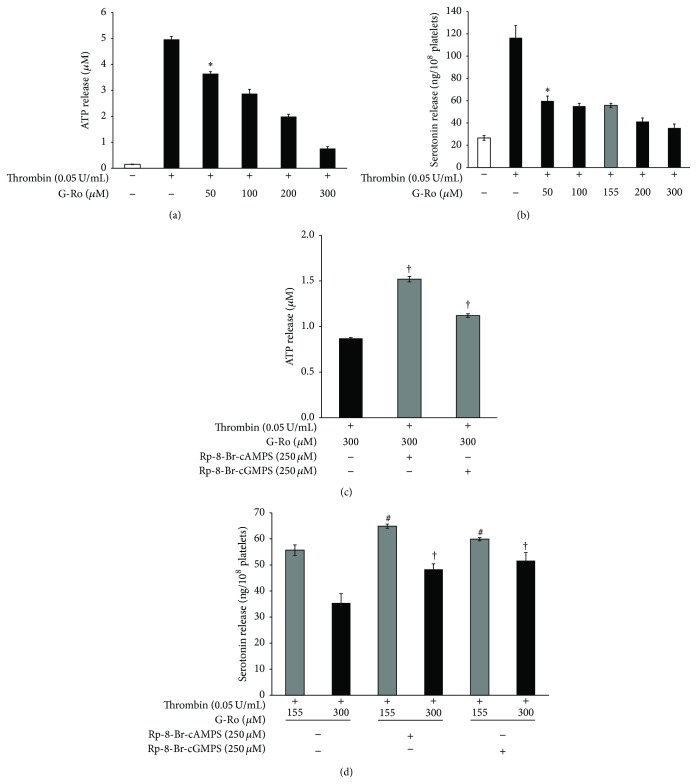
Effects of G-Ro on ATP and serotonin release. (a) Effects of G-Ro on ATP release in thrombin-activated platelets. (b) Effects of G-Ro on serotonin release in thrombin-activated platelets. (c) Effects of G-Ro on ATP release in the presence of A-kinase inhibitor (Rp-8-Br-cAMPS) and G-kinase inhibitor (Rp-8-Br-cGMPS). (d) Effects of G-Ro on serotonin release in the presence of A-kinase inhibitor (Rp-8-Br-cAMPS) and G-kinase inhibitor (Rp-8-Br-cGMPS). Determination of ATP and serotonin release was carried out as described in “Section 2.” The data are expressed as mean ± standard deviation (*n* = 4). ^*∗*^ *p* < 0.05 versus the thrombin-stimulated human platelets, ^#^ *p* < 0.05 versus the thrombin-stimulated human platelets in the presence of G-Ro (155 *μ*M), and ^†^ *p* < 0.05 versus the thrombin-stimulated human platelets in the presence of G-Ro (300 *μ*M).

Dense body contains nucleotides (ATP and ADP) and serotonin; these are released by collagen- or thrombin-elevated [Ca^2+^]_*i*_ and subsequently involve amplification of platelet activation [33–36]. Because G-Ro decreased thrombin-elevated [Ca^2+^]_*i*_ level, we investigated whether G-Ro is involved in inhibition of ATP and serotonin release. G-Ro (50 to 300 *μ*M) dose-dependently inhibited thrombin-induced ATP (Figure 8(a)) and serotonin release (Figure 8(b)). Next, we investigated whether the inhibition of ATP and serotonin release by G-Ro was dependent on cAMP/PKA-pathway and cGMP/PKG-pathway. G-Ro-downregulated (300 *μ*M) release of ATP and serotonin was significantly increased in the presence of PKA inhibitor Rp-8-Br-cAMPS and PKG inhibitor Rp-8-Br-cGMPS (Figure 8(c)). In addition, these inhibitors significantly elevated G-Ro-decreased (155, 300 *μ*M) serotonin release (Figure 8(d)).

### 3.8. Effects of G-Ro on p-Selectin Expression

**Figure 9:**
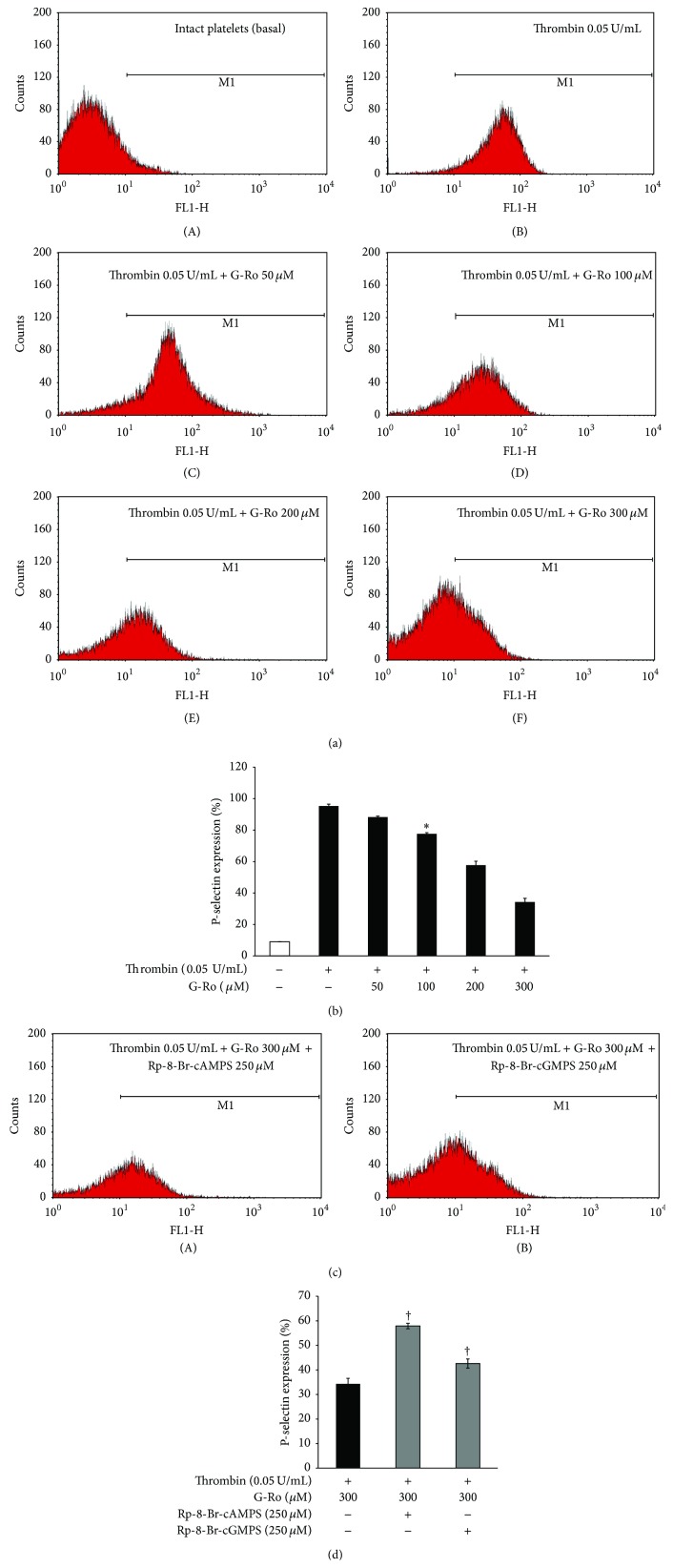
Effects of G-Ro on p-selectin expression. (a) The flow cytometry histograms on p-selectin expression. (A) Intact platelets (base); (B) thrombin (0.05 U/mL); (C) thrombin (0.05 U/mL) + G-Ro (50 *μ*M); (D) thrombin (0.05 U/mL) + G-Ro (100 *μ*M); (E) thrombin (0.05 U/mL) + G-Ro (200 *μ*M); (F) thrombin (0.05 U/mL) + G-Ro (300 *μ*M). (b) Effects of G-Ro on thrombin-induced p-selectin expression (%). (c) The flow cytometry histograms on p-selectin expression in the presence of A-kinase inhibitor (Rp-8-Br-cAMPS) and G-kinase inhibitor (Rp-8-Br-cGMPS). (A) Thrombin (0.05 U/mL) + G-Ro (300 *μ*M) + Rp-8-Br-cAMPS (250 *μ*M); (B) thrombin (0.05 U/mL) + G-Ro (300 *μ*M) + Rp-8-Br-cGMPS (250 *μ*M). (d) Effects of G-Ro on thrombin-induced p-selectin expression in the presence of A-kinase inhibitor (Rp-8-Br-cAMPS) and G-kinase inhibitor (Rp-8-Br-cGMPS) (%). Determination of p-selectin expression was carried out as described in “Section 2.” The data are expressed as the mean ± standard deviation (*n* = 4). ^*∗*^ *p* < 0.05 versus the thrombin-stimulated human platelets; ^†^ *p* < 0.05 versus the thrombin-stimulated human platelets in the presence of G-Ro (300 *μ*M).

Compounds in *α*-granule of platelets are known to be involved in inflammation, coagulation, and angiogenesis [36]; these are also Ca^2+^-dependently released by various platelet agonists. In particular, p-selectin is released from *α*-granule and is reexpressed to the platelet surface [37] and subsequently is involved in inflammation by binding to the p-selectin glycoprotein ligand-1 receptor on monocyte [38]. Therefore, we investigated the effect of G-Ro on p-selectin expression from *α*-granule. Thrombin stimulated the expression of p-selectin (Figure 9(a)-(B)) as compared with that by unstimulated platelets (Figure 9(a)-(A)). However, G-Ro (50 to 300 *μ*M) dose-dependently inhibited thrombin-induced expression of p-selectin (Figures 9(a)-(C)~(F) and 9(b)). PKA inhibitor Rp-8-Br-cAMPS increased G-Ro-reduced (300 *μ*M) p-selectin expression (Figures 9(c)-(A) and 9(d)), but PKG inhibitor Rp-8-Br-cGMPS did not increase as compared with that by Rp-8-Br-cAMPS (Figures 9(c)-(B) and 9(d)).

## 4. Discussion

Thrombin-elevated [Ca^2+^]_*i*_ is involved in various cellular events to activate platelets, which leads to granule secretion and platelet aggregation. IP_3_ that is generated from PIP_2_ by agonists (i.e., thrombin, collagen, and ADP) mobilizes [Ca^2+^]_*i*_ from Ca^2+^ store DTS* via* IP_3_RI. Depletion of the intracellular Ca^2+^ store by IP_3_ is known to connect the influx of extracellular Ca^2+^, which is stimulated by PI3K and ERK [14–18]. Even though G-Ro inhibited thrombin-elevated [Ca^2+^]_*i*_, it is not unknown if G-Ro inhibited the production of IP_3_ *via* inhibition of phospholipase C*β*. The Ca^2+^-antagonistic reaction by cAMP and cGMP is mediated by PKA/IP_3_RI and PKG/IP_3_RI phosphorylation pathways, respectively. Even if G-Ro would not inhibit PLC-*β* activity and IP_3_ production in thrombin-activated human platelets, if G-Ro that elevates the level of cAMP stimulates IP_3_RI (Ser^1756^) phosphorylation in thrombin-activated human platelets, this is a clear evidence that G-Ro is involved in inhibition of [Ca^2+^]_*i*_ mobilization from DTS* via* cAMP/PKA/IP_3_RI (Ser^1756^) phosphorylation pathway. In this report, we confirmed that G-Ro inhibited [Ca^2+^]_*i*_ mobilization* via* IP_3_RI (Ser^1756^) phosphorylation by cAMP/PKA, which is supported from the result that cAMP inhibitor Rp-8-Br-cAMPS inhibited G-Ro-elevated phosphorylation of IP_3_RI (Ser^1756^) in thrombin-induced human platelet aggregation; if not so, A-kinase inhibitor Rp-8-Br-cAMPS would not increase G-Ro-decreased [Ca^2+^]_*i*_ mobilization in thrombin-induced human platelet aggregation. It is well established that PI3K and ERK are involved in influx of extracellular Ca^2+^ across* PM*. Because Fura 2-loaded washed platelets were stimulated by thrombin in the presence of CaCl_2_ (2 mM), thrombin-evoked [Ca^2+^]_*i*_ resulted from the Ca^2+^ mobilization from DTS by IP_3_ and the Ca^2+^ influx across PM by PI3K or ERK. Because G-Ro activated the phosphorylation of IP_3_RI (Ser^1756^) and the dephosphorylation of ERK 2 (42 kDa), the inhibition of thrombin-elevated [Ca^2+^]_*i*_ by G-Ro is thought to result from both phosphorylation of IP_3_RI (Ser^1756^) and dephosphorylation of ERK 2 (42 kDa) by G-Ro. It is known that IP_3_ induces serotonin release from platelet dense body, which means that IP_3_ is involved in serotonin release by elevating [Ca^2+^]_*i*_ *via* IP_3_RI [39]. This reflects that G-Ro may be involved in inhibition of serotonin release by phosphorylating IP_3_RI (Ser^1756^).

A lot of agonists such as collagen, thrombin, and ADP increase [Ca^2+^]_*i*_ to phosphorylate Ca^2+^/calmodulin-dependent myosin light chain (20 kDa), which is involved in granule secretion such as ATP and serotonin [8, 9] to intensify platelet aggregation. It is known that G-Ro does not reduce collagen-elevated [Ca^2+^]_*i*_ level [40]; however, in this study, we confirmed that G-Ro decreases thrombin-elevated [Ca^2+^]_*i*_ level by stimulating the cAMP-dependent phosphorylation of IP_3_RI (Ser^1756^) and dephosphorylation of ERK 2 (42 kDa). Therefore, it is thought that the inhibition of ATP and serotonin secretion by G-Ro resulted from the inhibition of IP_3_RI-mediated [Ca^2+^]_*i*_ mobilization, and ERK 2-mediated (42 kDa) Ca^2+^ influx.

Platelet aggregation is generated at site of vascular wall injury and is involved in the formation of thrombus. During the formation of thrombus, platelets release cell growth proteins such as platelet-derived growth factor (PDGF) and vascular endothelial growth factor (VEGF) in *α*-granule [41, 42]. It is well established that PDGF and VEGF induce the proliferation of fibroblast, vascular smooth cells, and epithelial cells and subsequently enhance the rate of atherosclerosis lesion progression [43–47]. The progression of atherosclerosis is strongly induced by inflammatory cell such as monocyte/macrophage and neutrophil [48]. Although G-Ro has antiplatelet effects, if G-Ro does not inhibit inflammation by leukocyte, the progression of atherosclerosis lesion would be generated at site of vascular wall injury, and a question for antiplatelet effects of G-Ro might be raised. ATP and serotonin released from dense body are known to be involved in amplification of platelet aggregation [33–36], and p-selectin released from *α*-granule is known to be involved in causing of inflammation [36–38]. Because G-Ro inhibited the release of ATP, serotonin, and p-selectin, it is thought that G-Ro may inhibit aggregation-amplification and inflammation. In real, G-Ro is known to have anti-inflammatory activity* in vivo* and* in vitro* [49, 50]. It is well reviewed that ginsenosides have anti-inflammatory effects by inhibiting the production of various proinflammatory mediators (i.e., PGE_2_ and NO) [51]. With regard to the composition of G-Ro, Sanada et al. [19] reported that G-Ro is contained in* Panax ginseng*, and Choi [20] very well reviewed that G-Ro (0.045 w/w %) is contained in* Panax ginseng*, but not in* Panax notoginseng* (Sanchi ginseng). It has been reported that G-Ro inhibited arachidonic acid-induced platelet aggregation and fibrin formation* in vitro* [52] and activated fibrinolysis, indicating the inhibition of fibrin thrombi* ex vivo* [53].

Considering these previous reports [49–53], it is thought that G-Ro may have antithrombotic and antiatherosclerotic effects without generation of inflammation and progression of atherosclerotic lesion at site of vascular wall injury. Therefore, G-Ro is highlighted as a nontoxic antiplatelet compound together with effect that G-Ro did not affect LDH leakage.

In conclusion, the most important result of this study is that G-Ro cAMP-dependently phosphorylates IP_3_RI (Ser^1756^) and dephosphorylates ERK 2 (42 kDa) to reduce thrombin-elevated [Ca^2+^]_*i*_ level, which contributed to attenuating the release of ATP, serotonin, and p-selectin. In addition, G-Ro could be clinically applied to the prevention of platelet-mediated thrombosis. Therefore, it is thought that G-Ro may represent a useful tool in the therapy and prevention of vascular diseases associated with platelet aggregation.

## Figures and Tables

**Table 1:** Effects of Rp-8-Br-cAMPS and Rp-8-Br-cGMPS on [Ca^2+^]_*i*_ mobilization.

	[Ca^2+^]_*i*_ (nM)	Increase (%)
G-Ro (155 *μ*M) + thrombin (0.05 U/mL)	160.3 ± 8.1	—
G-Ro (155 *μ*M) + Rp-8-Br-cAMPS (250 *μ*M) + thrombin (0.05 U/mL)	326.3 ± 9.0	103.6^(1)^
G-Ro (155 *μ*M) + Rp-8-Br-cGMPS (250 *μ*M) + thrombin (0.05 U/mL)	202.6 ± 4.6	26.4^(2)^

G-Ro (300 *μ*M) + thrombin (0.05 U/mL)	140.1 ± 1.8	—
G-Ro (300 *μ*M) + Rp-8-Br-cAMPS (250 *μ*M) + thrombin (0.05 U/mL)	260.3 ± 9.9	85.8^(1)^
G-Ro (300 *μ*M) + Rp-8-Br-cGMPS (250 *μ*M) + thrombin (0.05 U/mL)	191.8 ± 7.3	36.9^(2)^

Data were from Figures 4(b) and 4(c). (1) Increase (%) = [(G-Ro + thrombin + Rp-8-Br-cAMPS) − (G-Ro + Thrombin)]/(G-Ro + thrombin) × 100, (2) increase (%) = [(G-Ro + thrombin + Rp-8-Br-cGMPS) − (G-Ro + thrombin)]/(G-Ro + thrombin) × 100.
